# Lifestyle Disease Risk Factors in a North Indian Community in Delhi

**DOI:** 10.4103/0970-0218.69279

**Published:** 2010-07

**Authors:** Ananya Laskar, Nandini Sharma, Neelima Bhagat

**Affiliations:** Department of Community Medicine, Maulana Azad Medical College, New Delhi - 110 002, India

## Introduction

Chronic diseases, better known as non-communicable diseases (NCDs), account for 53% of the deaths and 44% of DALYs in India. Although these chronic diseases are highly prevalent in urban areas, they are inadequately detected.(1) Integrated disease surveillance project endeavors to conduct periodic surveillance of risk factors for chronic diseases using the WHO-STEPS approach.(2) This approach focuses core and expanded data on established risk factors of NCDs. The three levels of risk factor assessment include history of risk factors (Step 1), simple physical measurements (Step 2), and biochemical measurements (Step 3).(3) The purpose of this study was to assess the magnitude of the risk factors of chronic diseases in order to initiate steps for effective interventions.

## Materials and Methods

A community-based cross-sectional study was undertaken in the Feroz Shah Kotla (FSK) area from November 2007 to February 2008. This settlement is situated beside the famous FSK stadium in the Central District of Delhi. It has an adult population of 2500, mainly comprising middle class Punjabis. Many commercial and tertiary health care centers are strategically located within 2–5 km of its radius. A sample size of 619 was estimated based on the current prevalence of risk factors of NCDs in Delhi (10–30%), using 3% precision and 95% confidence interval. The area consists of five blocks A–E, and proportionate numbers of residents were selected from each block using the systematic random sampling method.

### Study instrument

The WHO Step 1 and 2 questionnaires were used which consisted of a set of core and expanded questions pertaining to tobacco intake, alcohol consumption, physical activity, and diet. The history of hypertension and diabetes documented by a physician was recorded along with the health-seeking behavior. As part of the preliminary study, anthropometry of only 186 (33%) of the participants was carried out. Informed consent was taken from all the participants.

### Statistical analysis

The data were analyzed in SPSS version 17 and the χ^2^test was applied to analyze the qualitative data.

## Results

In the present study, out of a total of 560 participants, majority, 350 (62.5%) were females as compared to 210 (37.5%) males. A total of 68% of the subjects had completed their secondary education and 25% were graduates or with higher degrees. Majority of the subjects belonged to either lower middle (49.3%) or upper middle income group (25.35%). A total of 49.2% of them were engaged in business, 16.4% were in government jobs, and 23.8% had retired from the service.

The prevalence of various risk factors is presented in Table 1. Although the past smoking rate was observed to be 31%, only 17.6% were currently smoking. A total of 50.7% males admitted that they occasionally indulged in social drinking and only 2.45% consumed alcohol daily.

**Table 1:** Prevalence of risk factors among the study subjects

Risk factor	Male *N*_1_=210 *n* (%)	Female *N*_2_=350 *n* (%)	Total *N*=560
Tobacco consumption			
C– Current smokers	37 (17.6)	0	37 (17.6)
Daily intake of cigarette (mean no. of cigarettes ± SD)	6.45±2.4	NA	6.45±2.4
Mean age of commencement	24.6±3.5	NA	24.6±3.5
Duration of years of smoking	32.4±4.8	NA	32.4±4.8
E – Past smokers	65 (30.9)		65 (30.9)
Use of smokeless products (sniffs/zarda)	19 (0.9)	5 (0.01)	24 (4.28)
Use of pan masala, gutka, etc.	53 (25.23)	32 (9.14)	85 (15.2)
Alcohol consumption			
C – Occasional consumption^#^	106 (50.47)	0	106 (50.47)
Daily consumption	5 (2.38)	NA	5 (2.38)
E – Largest drink consumed in the past 12 months			
1–4 drinks (peg)	117 (73.6)	0	
>5 drinks (peg)	86 (40.9)	0	
Mean age of initiation	19.45±3.5	NA	
Physical activity			
Moderate physical activity of 30 min at least 5 day/week	44 (20.9)	56 (16)	100 (17.8)
Median time spent in moderate activity at work (min)	20 (0-60)	30 (15-45)	
Brisk walking (min)	14.54±7.25	10.65±8.4	
Vegetable consumption (servings/day)			
>3 times	38 (18.1)	56 (16)	94 (16.8)
2 times	83 (39.5)	114 (32.6)	297 (53)
Once	79 (37.6)	180 (51.4)	259 (46.3)
Fruit consumption (no. of times/week)			
Daily	25 (11.9)	31 (8.8)	56 (1)
5–6/week	43 (20.47)	38 (10.8)	81 (14.5)
3–4/week	56 (26.6)	65 (18.6)	121 (21.6)
1-2/wk	81 (38.6)	200 (57.2)	281 (50.2)
Rarely/never	5 (2.3)	16 (4.5)	21 (3.75)

C=core; E=expanded questions

The daily intake of fruits in a typical week was only 1%. The cooking oil used routinely by 96% was refined vegetable oil with only occasional use of ghee or vanaspati. Among the vegetable oils “Fortune” brand of soya oil was the most popular (78%). Alterations in the use of fats was more marked in families having one or more members suffering from any of the lifestyle diseases.

The overall prevalence of known cases of hypertension was 36.9% and that of diabetes was 10.53%. The health-seeking behavior was poor as evident from Table 2. Men were more particular about their routine check-ups such as weight or blood pressure measurements than women. Compliance was 91.5% to anti-diabetic treatment as against 47% to antihypertensive treatment. The mean BMI among women was higher (23.6 kg/m^2^) than men (22.7 kg/m^2^). Gender difference was conspicuous in their waist-hip ratios too.

**Table 2:** Prevalence of chronic disease among the study subjects

	Males (*N*=210) *n* (%)	Females (*N*=350) *n* (%)	Total (*N*=560) *n* (%)
Got BP measure in the past 12 months	134 (63.8)	146 (41.7)	280 (50%)*
*Hypertensives*	72 (34.28)	135 (38.57)	207 (36.9)
Advised salt restriction	64 (88)	110 (81.5)	174 (84.05)
Advised to lose weight	28 (38.8)	50 (37)	78 (37.6)
Advised to stop smoking	33 (45.8)	NA	33 (15.9)
Complying to antihypertensive medications	33 (45.8)	66 (48.8)	99 (47.8)
Got blood sugar tested in 12 months	114 (54.3)	75 (21.4)	189 (33.75)**
*Diabetics*	27 (12.83)	32 (9.14)	59 (10.53)
Regular medication for diabetes	24 (88.8)	30 (93.75)	54 (91.5)
Any special diet prescribed	12 (44.4)	16 (50)	28 (47.5)
Advised to lose weight	18 (66.6)	12 (31.3)	30 (50.8)
Anthropometry Total (*N*=186)	Males (*N*=92)	Females (*N*=94)	WHO cut-offs
*BMI*(kg/m^2^) Mean ± SD	22.68±2.02	23.6±2.21	23 kg/m^2^
Overweight *n* (%)	25 (27.17)	41 (43.61)	25 kg/m^2^
Obese *n* (%)	11 (11.95)	14 (14.8)	
*Waist–hip ratio*	1.03±0.27	1.058±0.29	0.8 for females and 1 for males
Mean ± SD	36 (39.13)	80 (86.9)	
Abnormal *n*(%)			

* *P*-value <0.001. The difference was significant between males and females with χ^2^=25.58, OR=2.46(1.71–3.56);

** The difference was significant at *P*<0.001, χ^2^=63.26, OR=4.35 (2.95–6.44)

## Discussion

The prevalence of risk factors for NCDs was high in the present study. According to NFHS-3 estimate, 57% males and 10.9% females consume tobacco.(4) Although the present study reported current smoking prevalence of 17.6% which is well below the national average, the proportion of subjects using pan-masala or gutka was high.

The occasional alcohol consumption in the present study was 50.5% with the age of initiation being 19 years, which is higher than NFHS-III estimate of 32%.(4) Delhi has witnessed a steadily declining age of initiation of alcohol during the past two decades, which is an alarming sign.(5) Only 17.8% were engaged in moderate physical activity despite the availability of parks and open space. Results were in agreement with another study in Delhi.(6)

Punjabi cuisine is famous for lavish use of cream and ghee but deviations in the routine food habits were observed, particularly in the use of refined “soya-oil,” which is a positive change. The vegetable and fruit intake was much below the WHO recommended standards of 400-500 g per day,(7) despite ease of availability and affordability. In our study, the prevalence of hypertension was 36.9% out of which 47% were compliant to treatment which is a matter of great concern. A substantial number of hypertensives and diabetics had not been advised any lifestyle modifications which is a missed opportunity. Another key finding of our study was that the anthropometric risk factors as well as the health-seeking behavior were poorer among the women. Similar results have been observed by a study conducted by AIIMS in Ballabhgarh.(8)

## Conclusion

The present study highlights a high prevalence of risk factors for chronic diseases and poor health-seeking behavior, despite accessibility and affordability to all levels of health care. Preventive health interventions need to be tailor-made according to community and gender needs. The focus should not only be on individual health education but also on health promotive lifestyles
